# Study on Correlation between Type 2 Diabetes and No-Reflow after PCI

**DOI:** 10.1155/2022/7319277

**Published:** 2022-03-17

**Authors:** Su-Rui Zhao, Rui Huang, Fang Liu, Ya Li, Yue Gong, Jun Xing

**Affiliations:** ^1^Department of Cardiology IV, Cangzhou Central Hospital, Cangzhou, 061001 Hebei Province, China; ^2^Department of Cardiology II, Cangzhou Central Hospital, Cangzhou, 061001 Hebei Province, China; ^3^Department of Gynecology and Obstetrics, Cangzhou Peace Hospital, Cangzhou, 061001 Hebei Province, China

## Abstract

Diabetes, a serious chronic disease globally, is often complicated with cardiovascular diseases for which percutaneous coronary intervention (PCI) is the mainstay. The no-reflow rate of diabetic patients after PCI is 2-4 times higher than that of nondiabetic patients, yet the specific mechanism is still unclear. This study was designed to investigate the correlation between the duration of diabetes, preoperative blood glucose level, coronary angiographic blood flow, coronary artery stenosis level, and no-reflow after PCI. A total of 131 patients with type 2 diabetes who underwent PCI in our hospital from 2019 to 2020 were divided into control group and observation group. The disease duration, preoperative blood glucose level, coronary angiographic blood flow, and coronary artery stenosis level of the two groups were calculated. There were differences in the duration of diabetes between the two groups; the blood glucose level of the control group was about 3.8%, which was lower than 5.8% of the observation group; the thrombolysis in myocardial infarction (TIMI) value of the control group was 18.46 ± 4.6, which was lower than 20.67 ± 3.9 of the observation group; The degree of coronary stenosis in the control was 63% ± 2%, which was lower than 76% + 3% in the observation group. Binary logistic stepwise regression analysis was performed on these indicators and no-reflow after PCI to explore the correlation between these indicators and no-reflow after PCI in diabetic patients. The study found that the diabetes duration, higher preoperative blood glucose level, coronary angiography blood flow, and coronary artery were positively associated with no-reflow after PCI.

## 1. Introduction

Diabetes is a globally serious chronic disease, with approximately 425 million patients worldwide, and 114 million adults in China [1, 2]. Studies have shown that diabetes patients aged over 50 years are susceptible to coronary heart disease with 12%-31.7% probability [3]. The United States spends as much as 348 billion dollars on diabetes treatment research every year, of which cardiovascular complications of diabetes accounts for 1/4, indicating that the cardiovascular disease (CVD) complicated by diabetes has imposed substantial burden to patients and society [4]. At present, CVD tops the causes of death among urban and rural residents in China, accounting for 40% [5]. Percutaneous coronary intervention (PCI) is commonly applied in the treatment of CVD, and it alleviates the patient's pain and improves the prognosis [6]. However, bare-metal stent (BMS) is prone to restenosis in the stent following PCI, leading to nonreflow. The incidence of such event is as high as 16%-44%, and it is the most daunting obstacle for PCI [7]. It is proved that the incidence of in-stent restenosis in diabetes patients after PCI is 2-4 times higher than that of nondiabetic patients [8, 9], but the exact mechanism remains unclear. To this end, this study aims at exploring the correlation between disease duration, preoperative blood glucose levels, coronary angiographic blood flow conditions, coronary stenosis levels, and no-reflow after PCI.

## 2. Materials and Methods

### 2.1. Subjects

**Figure 1:**
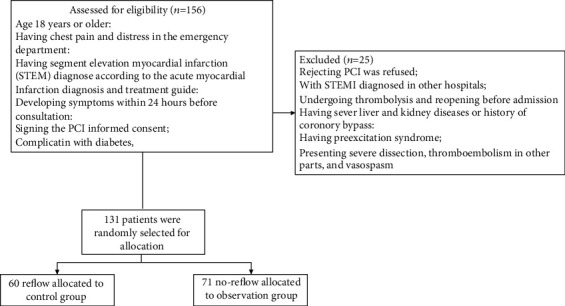
Flowchart of enrollment.

A total of 131 patients with type 2 diabetes who underwent PCI in our hospital from 2019 to 2020 were divided into 2 groups: control group (reflow, *n* = 60) and observation group (no-reflow, *n* = 71). The flowchart of enrollment was shown in Figure 1. Before enrollment, the general data including demographic characteristics, medical history, and blood pressure were collected by doctors. The diagnostic criteria for acute myocardial infarction (AMI) were in accordance with the Diagnostic and Therapeutic Guidelines for AMI developed by the Cardiovascular Branch of the Chinese Medical Association and the Editorial Board of Chinese Journal of Cardiovascular Disease and Chinese Journal of Circulation [10]. The diabetes is diagnosed according to the 2017 American Diabetes Association Diabetes Diagnosis and Treatment Standard.

### 2.2. Inclusion and Exclusion Criteria

Inclusion criteria are as follows: (1) patients aged 18 years or older; (2) patients had chest pain and distress in the emergency department; (3) patients had segment elevation myocardial infarction (STEMI) diagnosed according to the Acute Myocardial Infarction Diagnosis and Treatment Guide; (4) patients developed symptoms within 24 hours before consultation; (5) patients signed the PCI informed consent; and (6) patients complicated with diabetes. Exclusion criteria are as follows: (1) patients rejected PCI; (2) patients with STEMI diagnosed in other hospitals; (3) patients underwent thrombolysis and reopened before admission; (4) patients had severe liver and kidney diseases, had no previous left main disease, or had a history of coronary bypass; (5) patients had preexcitation syndrome; and (6) patients presented severe dissection, thromboembolism in other parts, and vasospasm.

### 2.3. Institutional Review Board Statement

This study was approved by the Ethics Committee of Cangzhou Central Hospital and was in accordance with the Helsinki Declaration [11], all participants and their families signed informed consent forms before enrollment.

### 2.4. PCI Procedure

All patients were punctured by the radial artery or femoral artery within 12 hours of onset and underwent coronary angiography and PCI treatment. Coronary angiography was used to determine the location, stenosis, or occlusion range of the infarcted vessel. According to the specific circumstances, the lesion site was superselected; and thrombus aspiration, balloon dilation, or stent placement was performed. Residual stenosis ≤ 10% was used as the standard to judge successful vascular opening. After the operation, the catheter was withdrawn, and the patients who underwent radial artery puncture were locally compressed to stop bleeding for 6 hours, and those who underwent femoral artery puncture were locally compressed to stop bleeding for 24 hours. Before PCI, ticagrelor (AstraZeneca Pharmaceutical Co., Ltd., specification: 90 mg) 180 mg and aspirin (Bayer Health Care Company, specification: 100 mg) 300 mg were administered via chewing. Postoperatively, ticagrelor, 90 mg/time, 2 times/d and aspirin 100 mg/time, 1 time/d were administered orally until 1 year after surgery. Some patients with severe myocardial ischemia received intravenous infusion of tirofiban (Lunan Pharmaceutical Factory, specification: 12.5 mg) for 3 days after operation, and beta-blocker Betaloc (AstraZeneca Pharmaceutical Co., Ltd., specification: 25 mg), ACEI/ARB irbesartan (Zhejiang Huahai Pharmaceutical Co., Ltd., specification: 75 mg), or benazepril (Shenzhen Xinlitai Pharmaceutical Co., Ltd., specification: 5 mg) were orally administered for a long run.

### 2.5. Criteria of No-Reflow

The coronary angiographic blood flow classification was applied by two senior cardiac interventional experts to determine the no-reflow phenomenon. No-reflow means that although the occlusioned coronary artery has been opened after emergency PCI treatment after excluding vascular spasm, dissection, and other related factors, there is no effective blood reperfusion in the ischemic myocardial tissue, that is, the coronary blood flow is slowed down or there is no blood flow. TIMI blood flow grading: TIMI 0 indicates that there is no forward blood flow outside the occlusion point. TIMI 1 indicated weak forward blood flow at the distal end of the coronary artery and incomplete filling at the distal end of the coronary bed. TIMI 2 represents complete distal coronary artery filling with delayed or slow forward flow. TIMI 3 indicates normal blood flow with the distal coronary artery fully filled. TIMI blood flow was recorded in the last frame of coronary angiography after stent implantation during PCI, and TIMI blood flow < level 2 was judged as no cardiac reflow; otherwise, it was considered as cardiac reflow.

The diabetes time, preoperative blood glucose levels, coronary angiographic blood flow, and coronary CT stenosis levels were monitored and compared. The number of patients with no-reflow and the number of deaths were compared between the two groups.

### 2.6. Statistical Analysis

The sample size was determined by power analysis (statistical power of 0.80, *α* of 0.05) with a moderate effect size (0.25) and an allocation ratio of 1 using the G∗Power, yielding 40 patients per group for a total of 80 patients. More than 40 patients were enrolled in each group. Primary and secondary outcome continuous variables were analyzed using the Student *t*-test. The Mann–Whitney *U* test was used when data were not equally distributed. Nominal variables were analyzed using *χ*^2^ tests. Binary logistic stepwise regression analysis was performed on these indicators and no-reflow after PCI to explore the correlation between these indicators and no-reflow after PCI in diabetic patients. Receiver Operating Characteristic (ROC) curve was conducted and Area Under the Curve (AUC) was calculated. All *p* values were 2-tailed, and the significance level was set at 0.05. All analyses were performed using the SPSS version 25.0 software.

## 3. Results

### 3.1. Clinical Data of Participants

There were no significant differences in age, gender, family history of coronary heart disease, history of cerebrovascular disease, history of angina pectoris, systolic blood pressure, diastolic blood pressure, pulse pressure, etc. between the two groups (Table 1), indicating that the baseline information in the two groups at the initial assessment was well balanced.

### 3.2. Correlation between the Duration of Type 2 Diabetes and the Incidence of No-Reflow

**Figure 2:**
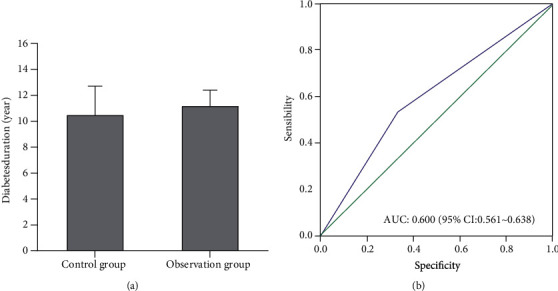
Correlation between the duration of type 2 diabetes and the incidence of no-reflow.

There were differences in the duration of diabetes between the two groups, but the differences were not statistically significant. The duration of disease in the control group was 10 ± 2.8 years, and the duration of observation group was 11.2 ± 1.9 years (Figure 2(a)). Correlation analysis showed that duration of diabetes was positively correlated with no-reflow after PCI (Figure 2(b)).

### 3.3. Correlation between Blood Glucose Levels and the Incidence of No-Reflow

**Figure 3:**
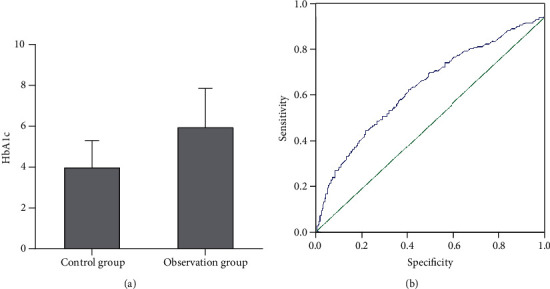
Correlation between blood glucose levels and the incidence of no-reflow.

HbA1c reflects the blood glucose level of patients, the blood glucose level of the control group was about 3.8%, which was lower than 5.8% of the observation group (Figure 3(a)). Correlation analysis revealed that the blood glucose level was positively correlated with no-reflow after PCI (Figure 3(b)).

### 3.4. Correlation between Coronary Blood Flow and Incidence of No-Reflow

**Figure 4:**
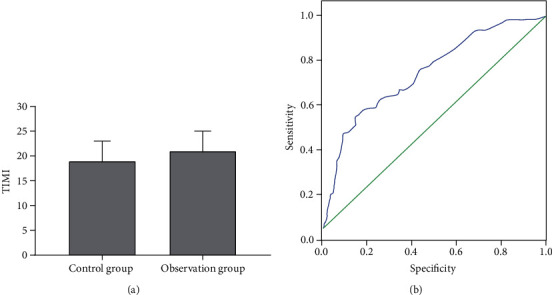
Correlation between coronary blood flow and incidence of no-reflow.

The thrombolysis in myocardial infarction (TIMI) value could accurately reflect the coronary blood flow of the patient, and the higher the value, the slower the blood flow. As shown in Figure 4(a), the TIMI value of the control group was 18.46 ± 4.6, which was lower than 20.67 ± 3.9 of the observation group. The correlation between the TIMI value and the case of no-reflow after PCI showed TIMI value and the no-reflow was positively correlated (Figure 4(b)).

### 3.5. Correlation between the Degree of Coronary Stenosis and the Incidence of No-Reflow

**Figure 5:**
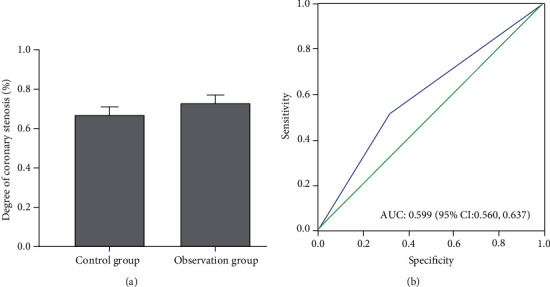
Correlation between the degree of coronary stenosis and the incidence of no-reflow.

There is also a correlation between the degree of coronary stenosis and the incidence of no-reflow after PCI. The degree of coronary stenosis in the control was 63% ± 2%, which was lower than 76% + 3% in the observation group. The correlation between the degree of coronary stenosis and the incidence of no reflow after PCI showed that the degree of coronary stenosis was positively correlated with the incidence of no reflow after PCI, i.e., the higher the degree of coronary stenosis, the easier the occurrence of no-reflow (Figure 5).

## 4. Discussion

Diabetes causes metabolic disorder, atherosclerosis of large and medium blood vessels, and microvascular disease, and the prevalence of diabetes has been on a rise [12]. Therefore, patients with diabetes often complicate with coronary heart disease, and many severe coronary artery diseases also occur in multiple blood vessels and single blood vessels. PCI is a widely used treatment for cardiovascular disease, yet no-reflow might occur in diabetic patients after PCI [13, 14], and the correlation between these two remains unclear. Thus, the purpose of this study was to investigate the correlation between the diabetes course, preoperative blood glucose levels, coronary angiographic blood flow, coronary CT stenosis, and no-reflow after PCI.

The study found that the longer the diabetes duration, the higher the preoperative blood glucose level, the slower the coronary angiography blood flow, the narrower the coronary artery, and the more likely they were to have no-reflow after PCI. The longer the time of diabetes, the more serious the impact on cardiovascular complications thus more likely to induce no-reflow after PCI [15]. Insulin can mediate antilipolysis, but adipose tissue insulin resistance can reduce this effect, lowering lipoprotein lipase activity and further triggering hyperlipidemia. It serves as one of the main causes of dyslipidemia in diabetic patients and can aggravate coronary atherosclerosis [16]. After PCI, the application of the stent would destroy the coronary artery endothelial cell layer, cause platelet activation and aggregation, and promote the infiltration of leukocytes and monocytes to the injured site [17]. Excessive platelets and some inflammatory factors can induce macrophages to phagocytose and clear cellular debris from damaged cells. These processes further stimulate the migration and proliferation of resting vascular smooth muscle cells and endothelial cells, which may further narrow coronary artery [18–22], and lead to no-reflow after PCI. Consistently, our study showed that the longer the duration of diabetes, the higher the lipid concentration, which leads to narrower coronary arteries, reduces blood flow, and induces no-reflow after PCI. Moreover, in our study, patients with high HbA1c were found to have a higher incidence of no-reflow after PCI. HbA1c concentration and blood glucose concentration are positively correlated; this indicator has become the gold standard for judging diabetes control, due to its accurate reflection of the average level 8-12 weeks before taking the blood [23]. To our knowledge, insulin resistance in patients with diabetes causes metabolic disorders such as chronic hyperglycemia, which in turn affects vascular endothelial metabolism [24]. This can also lead to the migration and proliferation of vascular smooth muscle intimal cells, further narrowing coronary arteries [25]. Overall, the blood glucose level of diabetic patients would also induce no-reflow after PCI. After analysis, the following can be attributed to the increase of probability of nonrecurrence. The longer the course of type 2 diabetes, the longer the development time of vascular endothelial injury and oxidative stress response caused by hyperglycemia, and the more serious the harm, the more difficult the recanalization of vessels during PCI, and the higher the risk of nonrecurrence [26]. Patients who do not follow hypoglycemic treatment are more likely to suffer cardiovascular and cerebrovascular damage due to poor blood glucose level control, which aggravates the condition and increases the probability of nonrelapse [27]. Previous study has confirmed that IRI > 2.5 is an important marker of insulin resistance, and insulin insensitivity means that the body's ability to regulate blood glucose is weakened, and the harm caused by hyperglycemia is more serious, and the incidence of nonrelapse increases significantly [28]. PCI is inconvenient for patients with proximal coronary artery embolism, which is easy to cause damage to the vascular wall, difficult to recover hemodynamics, and easy to cause no recurrence due to vascular endothelial injury and inflammatory reaction [29]. Therefore, the above factors increase the probability of nonrecurrence during PCI in AMI patients with T2MD to a certain extent.

## 5. Conclusion

In summary, the prevalence of no-reflow in patients with diabetes after PCI is affected by multiple factors. The duration of the disease, preoperative blood glucose level, coronary blood flow, and coronary stenosis were positively correlated with the incidence of no-reflow after PCI in patients with diabetes.

## 6. Study Limitations

However, our study has certain limitations, i.e., it was a single-centered study with relatively small sample size which may result in biased results and conclusions. The prevalence of no-reflow in patients with diabetes mellitus after PCI is a complex disorder, in which multiple factors are involved. Therefore, more cases needed to be enrolled to explore the influence of multiple factors on the incidence of no-reflow in patients with diabetes mellitus after PCI. Still, some other relevant factors should be considered in the future study, including delay in procedure and local protocol.

## Figures and Tables

**Table 1:** The comparison of baseline information.

	Overall (*n* = 131)	Control group (*n* = 60)	Observation group (*n* = 71)	*t*/*χ*2	*p*
Age	64.73 ± 5.06	64.25 ± 4.81	65.13 ± 5.26	0.992	0.323
Male	71	31	40	0.286	0.593
Female	60	29	31		
SYNTAX score	15.15 ± 5.96	14.35 ± 6.23	15.82 ± 5.68	1.412	0.160
CHD family history	31	14	17	0.007	0.934
Cerebrovascular disease history	48	21	27	0.128	0.720
Systolic blood pressure	129.16 ± 23.15	129.96 ± 23.31	128.49 ± 23.16	0.361	0.719
Diastolic blood pressure	77.82 ± 14.35	78.02 ± 14.91	77.66 ± 13.97	0.143	0.887
Pulse pressure	51.33 ± 16.32	51.94 ± 16.09	50.82 ± 16.61	0.390	0.697

## Data Availability

The datasets used during the present study are available from the corresponding author upon reasonable request.
